# Development and validation of a nomogram model for predicting cardiac autonomic neuropathy in patients with diabetes

**DOI:** 10.3389/fendo.2026.1831010

**Published:** 2026-06-05

**Authors:** Binhui Jia, Zhuyi Jiang, Xuexian Wen, Mingming Yang, Jun Wang

**Affiliations:** 1 Department of Endocrinology, Shenzhen People’s Hospital, The Second Clinical Medical College, Jinan University, Shenzhen, China; 2 Department of Endocrinology, Shenzhen People’s Hospital (The First Affiliated Hospital, Southern University of Science and Technology, The Second Clinical Medical College, Jinan University), Shenzhen, China; 3 Department of Ophthalmology, Shenzhen People’s Hospital (The First Affiliated Hospital, Southern University of Science and Technology, The Second Clinical Medical College, Jinan University), Shenzhen, China

**Keywords:** diabetic cardiac autonomic neuropathy, early screening, machine learning, nomogram, prediction model, type 1 and type 2 diabetes, web-based calculator

## Abstract

**Background:**

Diabetic Cardiac Autonomic Neuropathy (DCAN), a critical yet frequently underdiagnosed microvascular complication, is associated with increased mortality. Standard cardiovascular autonomic reflex tests (CARTs) are complex and time-consuming, hindering their widespread use in routine screening in clinical settings. This study aimed to develop and validate a predictive nomogram for DCAN in patients with diabetes using readily available clinical variables.

**Methods:**

We retrospectively analyzed the clinical data of 453 patients with type 1 or type 2 diabetes hospitalized at Shenzhen People’s Hospital between February 2022 and December 2025. The dataset was randomly divided into training (70%) and validation (30%) cohorts. Key predictors were identified using a rigorous selection strategy that combined univariate analysis, least absolute shrinkage and selection operator (LASSO) regression, and multivariate logistic regression. Four candidate prediction models (Logistic Regression (LR), Random Forest, Extreme Gradient Boosting (XGBoost), and Light Gradient Boosting Machine (LightGBM) were constructed and evaluated for discrimination, calibration, and clinical utility. The optimal model was visualized as a nomogram and interactive web calculator.

**Results:**

The prevalence of DCAN in the study population was 45.0% (204/453). The following seven independent predictors were identified: a history of diabetic retinopathy (DR) or diabetic kidney disease (DKD), diabetes duration, age, heart rate (HR), fasting plasma glucose (FPG), and HbA1c. Among the algorithms tested, the LR model exhibited the most balanced performance in the validation cohort (area under the curve (AUC) = 0.838) with the highest sensitivity (77.0%) and was thus selected as the optimal prediction tool. Consequently, the LR model was transformed into a predictive nomogram. This nomogram demonstrated good calibration and potential clinical utility for individualized risk assessment.

**Conclusion:**

We successfully developed and validated a high-sensitivity prediction model for DCAN applicable to type 1 and type 2 diabetes. The developed visual nomogram and interactive web-based tool are cost-effective and user-friendly instruments that can facilitate early risk assessment and personalized clinical management.

## 1 Introduction

Diabetic cardiac autonomic neuropathy (DCAN), a serious yet frequently overlooked microvascular complication of diabetes mellitus, is characterized by damage to autonomic nerve fibers innervating the cardiovascular system, which disrupts heart rate (HR) control and hemodynamic regulation (1, 2). Epidemiological data indicate that DCAN prevalence ranges from 31% to 73% in patients with type 2 diabetes mellitus (T2DM) and 17% to 66% in those with type 1 diabetes mellitus (T1DM) (3). There is a strong association between DCAN and a spectrum of severe adverse cardiovascular and cerebrovascular outcomes, including silent myocardial infarction (MI), heart failure, malignant arrhythmia, ischemic stroke, and sudden cardiac death (4–7). Previous studies have reported that the 5-year mortality risk in patients with DCAN is approximately five times higher than that in individuals without autonomic dysfunction (1, 8, 9). However, the onset of DCAN is insidious and often lacks specific symptoms in its early stages, leading to substantial underdiagnosis in clinical practice (10, 11).

Cardiovascular autonomic reflex tests (CARTs) are the gold standard for diagnosing DCAN; however, these tests are complex, time-consuming, and require specialized equipment and trained personnel, hindering their implementation (12). Several patients miss the critical window for early detection and timely intervention, necessitating a simple and accessible screening tool to identify individuals at high risk of DCAN. To address the challenge of identifying cardiac autonomic neuropathy in patients with diabetes, previous studies have proposed a range of predictive approaches. These include logistic regression–based clinical risk scoring systems derived from routinely available clinical variables (13), logistic regression models incorporating parameters from standardized autonomic function tests such as the Ewing’s tests (14), as well as ensemble-based classification methods aimed at characterizing features associated with cardiac autonomic dysfunction (15). However, existing approaches vary considerably in terms of predictor selection, model complexity, and clinical applicability. Moreover, systematic comparisons between traditional statistical models and contemporary machine learning algorithms within a unified clinical framework remain limited, and the translation of predictive models into intuitive, clinically deployable tools has not been fully explored.

Developing a practical and reliable prediction model based on commonly available clinical variables may help identify those patients with diabetes who are also at a greater risk of presenting with DCAN, thereby facilitating earlier screening and management. In this study, we used data from a retrospective cohort of patients with both T1DM and T2DM to develop and validate a prediction model for DCAN. Four machine-learning algorithms—logistic regression, random forest, extreme gradient boosting (XGBoost), and light gradient boosting machine (LightGBM)—were constructed and systematically compared to determine the optimal model. Therefore, the aim of this study was to develop and validate a machine learning–based predictive model using readily available clinical variables to identify the presence of cardiac autonomic neuropathy in patients with diabetes.

## 2 Research design and methods

### 2.1 Study participants

**Figure 1:**
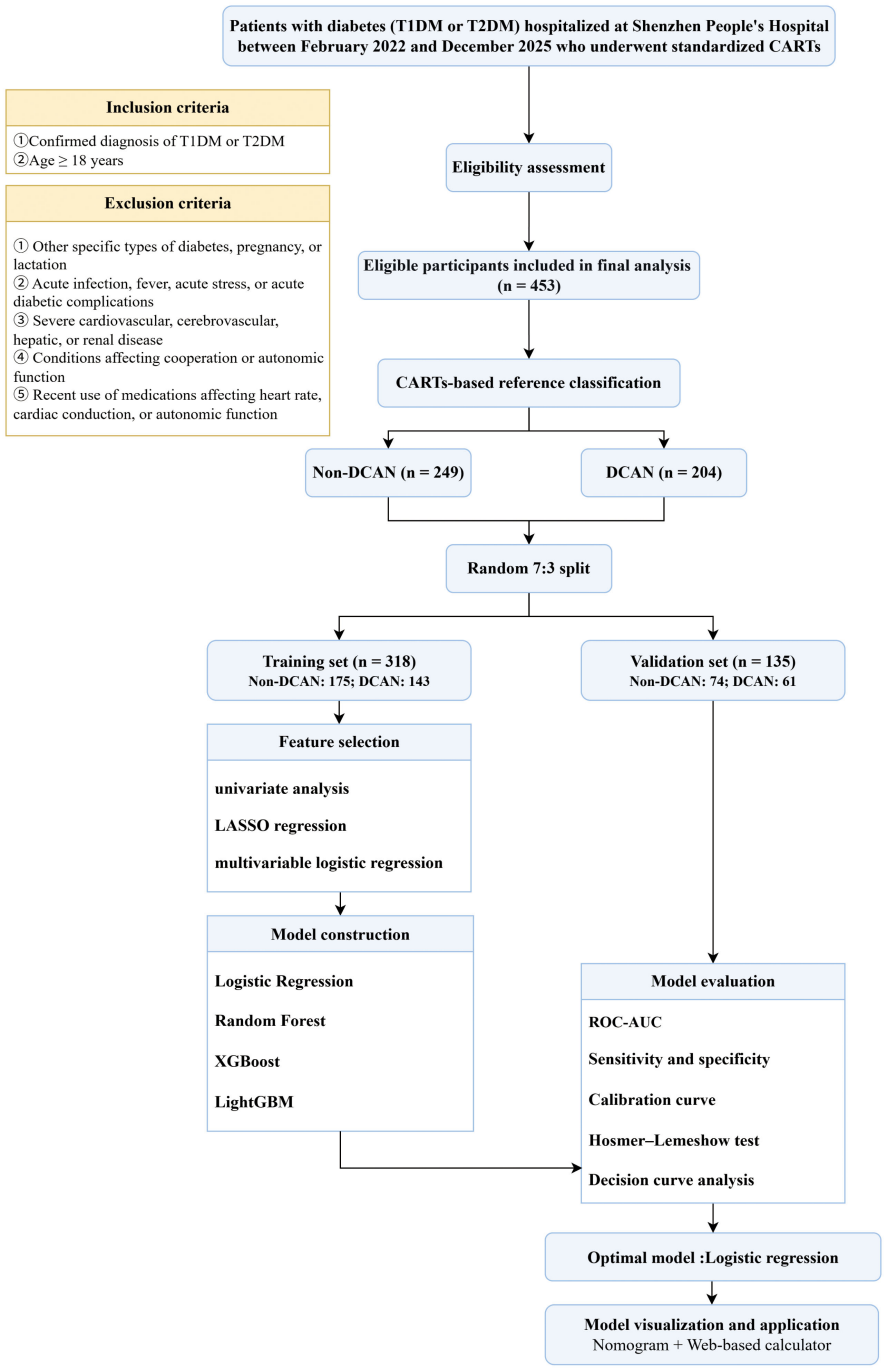
Study flow diagram.

We retrospectively analyzed 453 patients with diabetes admitted to the Department of Endocrinology at Shenzhen People’s Hospital between February 2022 and December 2025. The inclusion criteria were as follows: (1) A confirmed diagnosis of T1DM or T2DM (16); (2) Age ≥ 18 years. The exclusion criteria were as follows: (1) gestational diabetes or other specific types of diabetes; (2) acute infection, fever, or other acute stress conditions; (3) severe cardiovascular or cerebrovascular diseases, including acute coronary syndrome, New York Heart Association (NYHA) class III–IV heart failure, aortic dissection, acute ischemic stroke, or intracerebral hemorrhage; (4) severe hepatic or renal dysfunction (alanine aminotransferase or aspartate aminotransferase levels >3 times the upper limit of normal, or an estimated glomerular filtration rate <30 mL/min/1.73 m²); (5) acute or severe diabetic complications, such as diabetic ketoacidosis or hyperosmolar hyperglycemic state; (6) malignancy, severe trauma, or psychiatric disorders that could impair cooperation with standardized autonomic testing; (7) pregnancy or lactation; (8) conditions affecting the autonomic nervous system, such as overt thyroid dysfunction or neurodegenerative diseases; (9) use of medications known to influence HR, cardiac conduction, or autonomic function within the preceding two weeks, including β-adrenergic blockers, cardiac glycosides, non-dihydropyridine calcium channel blockers, systemic corticosteroids, or other agents deemed to interfere with standardized CARTs. The study flow diagram is shown in **Figure 1**.

All participants underwent CARTs. The study was conducted in accordance with the principles of the Declaration of Helsinki. This study was approved by the Ethics Committee of the Shenzhen People’s Hospital (approval no. LL-KY-2026076). All personal data were anonymized and de-identified prior to the analysis to protect patient privacy. The requirement for written informed consent was waived due to the study’s retrospective design.

### 2.2 Data collection

Clinical data were retrospectively extracted from the electronic medical records. The baseline demographic and clinical characteristics included age, sex, body mass index (BMI), diabetes duration, systolic blood pressure (SBP), diastolic blood pressure (DBP), pulse pressure (PP), history of hypertension, and history of microvascular complications [specifically diabetic retinopathy (DR) and diabetic kidney disease (DKD)]. Information on cardiovascular medication use, including angiotensin-converting enzyme inhibitors/angiotensin receptor blockers (ACEIs/ARBs) and dihydropyridine calcium channel blockers (CCBs), was also collected. Comprehensive laboratory assessments comprised fasting plasma glucose (FPG), glycated hemoglobin (HbA1c), fasting C-peptide, total cholesterol (TC), triglycerides (TG), high-density lipoprotein cholesterol (HDL-C), low-density lipoprotein cholesterol (LDL-C), aspartate aminotransferase (AST), alanine aminotransferase (ALT), serum albumin, serum creatinine (Scr), estimated glomerular filtration rate (eGFR), serum uric acid (SUA), and 25-hydroxyvitamin D [25(OH)D]. Hematological indices included white blood cell count, neutrophil count, lymphocyte count, monocyte count, and hemoglobin levels. Novel metabolic and inflammatory indices were calculated as follows: triglyceride-glucose (TyG) index = ln[TG (mg/dL) × FPG (mg/dL)/2]; systemic inflammation response index (SIRI) = (neutrophil count × monocyte count)/lymphocyte count.

### 2.3 Assessment and definition of DCAN

All participants underwent standardized CARTs in a controlled laboratory setting. The assessment for DCAN was based on the criteria proposed by the Toronto Diabetic Neuropathy Expert Group and endorsed by the American Diabetes Association, which served as the diagnostic reference in this study (17, 18).

#### 2.3.1 Patient preparation

To ensure standardization and reproducibility, participants were instructed to abstain from strenuous exercise for 24 hours before testing and to avoid smoking, alcohol, coffee, and tea within 2 hours before the assessment. Tests were conducted in a quiet environment with participants in a relaxed and awake state. Contraindications and factors that could affect autonomic assessment were reviewed before testing, and recent use of medications known to influence HR or autonomic function was controlled through the exclusion criteria.

#### 2.3.2 Cardiovascular autonomic reflex tests

The CARTs protocol comprised four standard tests:

1. Heart rate response to deep breathing: Subjects performed six cycles of deep breathing at a rate of 5 s inspiration and 5 s expiration. The result was expressed as the mean difference between the maximum and minimum HRs across the six cycles (beats/min).
2. HR response to the Valsalva maneuver (Valsalva ratio): Subjects exhaled into a mouthpiece maintained at a constant pressure of 40 mmHg for 15 s. The ratio was calculated as the longest R-R interval after the maneuver to the shortest R-R interval during the maneuver.
3. HR response to standing (30:15 ratio): Participants moved from a supine to a standing position within 5 s; the ratio of the R-R interval at the 30^th^ beat to that at the 15^th^ beat was recorded.
4. Blood pressure response to standing: SBP and DBP were measured in the supine position and after 3 minutes of active standing.

#### 2.3.3 Scoring and staging of DCAN

Each CART parameter was evaluated using the Ewing scoring system, in which results were classified as normal (0 points), borderline (1 point), or abnormal (2 points) according to predefined thresholds (19). The total Ewing score was calculated by summing the scores of the four tests, ranging from 0 to 8 points. The detailed scoring criteria for each CART parameter are provided in **Supplementary Table 1**.

In this study, Ewing scoring was used to quantify individual CART results, whereas the Toronto consensus framework was used to guide DCAN staging and diagnostic interpretation. Normal autonomic function was defined as no abnormal cardiovagal test result and a total Ewing score of 0–1. Early DCAN corresponded to mild autonomic abnormality, generally reflected by one abnormal cardiovagal test or a total Ewing score of 2–3. Definite DCAN was defined as at least two abnormal cardiovagal tests, corresponding to a total Ewing score of 4–6. Severe DCAN was defined as definite DCAN accompanied by orthostatic hypotension or marked orthostatic blood pressure abnormality, corresponding to a total Ewing score of 7–8.

#### 2.3.4 Binary outcome definition for prediction modeling

To develop a predictive model for identifying the presence of DCAN, the outcome was dichotomized. Patients classified as early, definite, or severe DCAN were assigned to the DCAN group, whereas those classified as normal were assigned to the non-DCAN group. Thus, CARTs-based severity classification served as the diagnostic reference framework, while the prediction model was designed to identify the presence rather than the severity of DCAN. Consequently, 204 and 249 patients were assigned to the DCAN and non-DCAN groups, respectively. Among the 204 patients with DCAN, 121 were classified as early, 65 as definite, and 18 as severe DCAN.

### 2.4 Sample size estimation

The adequacy of the available sample size was evaluated with reference to contemporary recommendations for the development of clinical prediction models, as proposed by Riley et al. (20). In this retrospective study, 204 outcome events (DCAN cases) were observed, and seven predictors were retained in the final model, resulting in an events-per-variable (EPV) ratio of approximately 29. This value exceeds commonly cited minimum EPV thresholds for logistic regression-based prediction models and supports the stability of model estimation.

### 2.5 Data preprocessing and dataset splitting

The dataset was randomly divided into a training set and an internal validation set at a ratio of 7:3 before imputation and model development. The training set was used for feature selection, model development, cross-validation, and hyperparameter tuning, whereas the internal validation set was reserved exclusively for final model evaluation. All model development procedures were conducted within the training set to reduce the risk of data leakage. After dataset splitting, missing data were handled using multiple imputation by chained equations with the mice package in R (n = 5). All variables with missing data had missing rates below 15%.

### 2.6 Feature selection and model building

A three-step feature selection strategy was implemented to identify independent predictors of DCAN in the training set. First, a univariate analysis was conducted on all collected clinical variables to screen for potential risk factors associated with DCAN. Variables with P < 0.05 were then entered into LASSO logistic regression to reduce model complexity and multicollinearity. Ten-fold cross-validation was performed to determine the optimal penalty parameter (λ) and variables with nonzero coefficients were retained. Finally, the variables selected by LASSO were entered into a multivariable logistic regression analysis to identify independent predictors of DCAN in patients with diabetes. The final predictors were used consistently for all candidate prediction models.

### 2.7 Machine learning algorithms and model construction

Based on the final selected predictors, four candidate prediction models were constructed: Logistic Regression (LR), Random Forest (RF), Extreme Gradient Boosting (XGBoost), and Light Gradient Boosting Machine (LightGBM). To optimize model performance, we tuned the hyperparameters of RF, XGBoost, and LightGBM in the training set using grid search with 5-fold cross-validation. The mean cross-validated area under the receiver operating characteristic curve (AUC) was used as the optimization criterion, and the final selected hyperparameters are provided in **Supplementary Table 2**.

### 2.8 Model comparison and visualization

To comprehensively assess the predictive performance, the discriminative abilities of all constructed models were compared using Receiver Operating Characteristic curves, AUCs, and 95% Confidence Intervals (CIs). Classification performance was further assessed using sensitivity, specificity, positive predictive value, negative predictive value, accuracy, and F1-score. The optimal cutoff value was determined using Youden’s index. Calibration was assessed using calibration curves and the Hosmer–Lemeshow goodness-of-fit test in both the training and internal validation sets. For the final nomogram, bootstrap resampling with 1,000 repetitions was used to generate bias-corrected calibration curves. Potential clinical usefulness was evaluated using decision curve analysis by comparing the net benefit of each model across a range of threshold probabilities with the treat-all and treat-none strategies.

The final model was selected based on the overall balance among discrimination, calibration, potential clinical usefulness, sensitivity, and interpretability. Because the model was intended for screening-oriented risk assessment rather than confirmatory diagnosis, sensitivity and interpretability were prioritized when model discrimination was comparable. This model was presented in two formats: a static nomogram constructed with the rms package in R and a dynamic web-based calculator developed with the Shiny application.

### 2.9 Statistical analysis

All statistical analyses were performed using Python (version 3.10.4) and R (version 4.5.1). The normality of continuous variables was assessed using the Kolmogorov-Smirnov test. Normally distributed continuous variables are reported as the mean ± standard deviation (SD) and compared using the Student’s *t*-test. Skewed continuous variables are presented as median (interquartile range) and compared using the Mann-Whitney *U* test. Categorical variables are expressed as frequencies and percentages [n (%)] and compared using the chi-square test or Fisher’s exact test, as appropriate. The 95% CIs for AUCs were calculated using bootstrap resampling. All statistical tests were two-sided, and a P < 0.05 was considered statistically significant.

## 3 Results

### 3.1 Baseline characteristics of the study population

In total, 453 patients with diabetes were included in this study: 288 males (63.6%) and 165 females (36.4%) with a combined median age of 57 years. Based on the CART assessment, 204 patients (45.0%) were diagnosed with DCAN (DCAN group), and 249 patients (55.0%) were classified as non-DCAN. Among the 204 patients with DCAN, 121 were classified as early DCAN, 65 as definite DCAN, and 18 as severe DCAN according to the CARTs-based reference framework. Baseline characteristics of the study population are summarized in **Table 1**.

**Table 1:** Baseline demographic and clinical characteristics between non-DCAN and DCAN groups.

Variables	Non-DCAN (n=249)	DCAN (n=204)	P-value
Male	163 (65.46)	125 (61.27)	0.410
Age, years	54.00 [48.00, 60.00]	61.00 [54.00, 66.00]	<0.001
BMI, kg/m²	24.30 [22.20, 26.53]	24.00 [22.27, 26.52]	0.489
Diabetes duration, years	7.00 [2.00, 14.00]	11.00 [7.00, 20.00]	<0.001
History of DR	30 (12.05)	91 (44.61)	<0.001
History of DKD	32 (12.85)	78 (38.24)	<0.001
Hypertension	101 (40.56)	101 (49.51)	0.070
ACEI/ARB use	70 (28.11)	65 (31.86)	0.444
Dihydropyridine CCB use	13 (5.22)	19 (9.31)	0.132
SBP, mmHg	127.00 [116.00, 135.00]	130.00 [116.00, 142.00]	0.033
DBP, mmHg	82.01 (10.41)	79.39 (10.35)	0.008
Pulse pressure, mmHg	44.00 [35.00, 52.00]	51.00 [39.00, 60.00]	<0.001
Heart rate, bpm	85.10 (11.39)	87.32 (11.88)	0.044
FPG, mmol/L	6.19 [5.18, 7.46]	7.16 [5.89, 9.42]	<0.001
HbA1c, %	8.10 [6.90, 9.80]	8.90 [7.50, 10.80]	<0.001
C-peptide, ng/mL	1.70 [0.97, 2.40]	1.65 [0.96, 2.40]	0.975
TC, mmol/L	4.62 [3.86, 5.37]	4.67 [3.67, 5.66]	0.526
TG, mmol/L	1.34 [0.93, 2.06]	1.48 [1.06, 2.26]	0.098
LDL-C, mmol/L	2.60 [1.90, 3.13]	2.67 [1.97, 3.41]	0.270
HDL-C, mmol/L	1.12 [0.97, 1.38]	1.13 [0.97, 1.35]	0.927
AST, U/L	20.00 [16.00, 25.00]	19.00 [16.00, 24.00]	0.385
ALT, U/L	21.00 [15.00, 29.00]	19.00 [14.00, 28.00]	0.207
Albumin, g/L	40.20 [38.00, 42.20]	40.15 [38.00, 42.80]	0.998
SCr, μmol/L	74.00 [63.00, 87.00]	72.00 [58.00, 92.00]	0.630
eGFR, mL/min/1.73m²	94.30 [83.18, 104.73]	90.81 [71.47, 103.41]	0.025
UACR, mg/g	6.09 [2.95, 14.96]	16.60 [6.55, 88.18]	<0.001
UA, μmol/L	351.00 [290.00, 409.00]	352.50 [292.75, 426.25]	0.714
25(OH)D, nmol/L	55.60 [43.00, 67.10]	52.70 [39.22, 65.44]	0.082
TyG index	8.85 [8.36, 9.40]	9.07 [8.61, 9.58]	0.001
SIRI	0.78 [0.53, 1.11]	0.90 [0.64, 1.32]	0.004

Data are presented as n (%), mean ± SD, or median [IQR].

ACEI/ARB, angiotensin-converting enzyme inhibitor/angiotensin receptor blocker; BMI, body mass index; CCB, calcium channel blocker; DCAN, diabetic cardiac autonomic neuropathy; DBP, diastolic blood pressure; DR, diabetic retinopathy; DKD, diabetic kidney disease; eGFR, estimated glomerular filtration rate; FPG, fasting plasma glucose; HbA1c, glycated hemoglobin; HDL-C, high-density lipoprotein cholesterol; LDL-C, low-density lipoprotein cholesterol; PP, pulse pressure; SBP, systolic blood pressure; SCr, serum creatinine; SIRI, systemic inflammation response index; TC, total cholesterol; TG, triglyceride; TyG, triglyceride-glucose index; UA, uric acid; UACR, urinary albumin-to-creatinine ratio; 25(OH)D, 25-hydroxyvitamin D.

Patients in the DCAN group were significantly older and had a diabetes duration that was longer than that of those in the non-DCAN group. The prevalence of DR and DKD was significantly higher in the DCAN group. Furthermore, patients with DCAN exhibited significantly elevated levels of PP, HR, FPG, HbA1c, urinary albumin-to-creatinine ratio (UACR), TyG index, and SIRI (all P < 0.05). However, no statistically significant differences were observed in sex distribution, BMI, hypertension prevalence, the use of ACEIs/ARBs or dihydropyridine calcium channel blockers, lipid profiles (TC, TG, LDL-C, and HDL-C), or liver function indices (AST and ALT) between the two groups (P > 0.05 for all comparisons).

### 3.2 Selection of predictors

**Figure 2:**
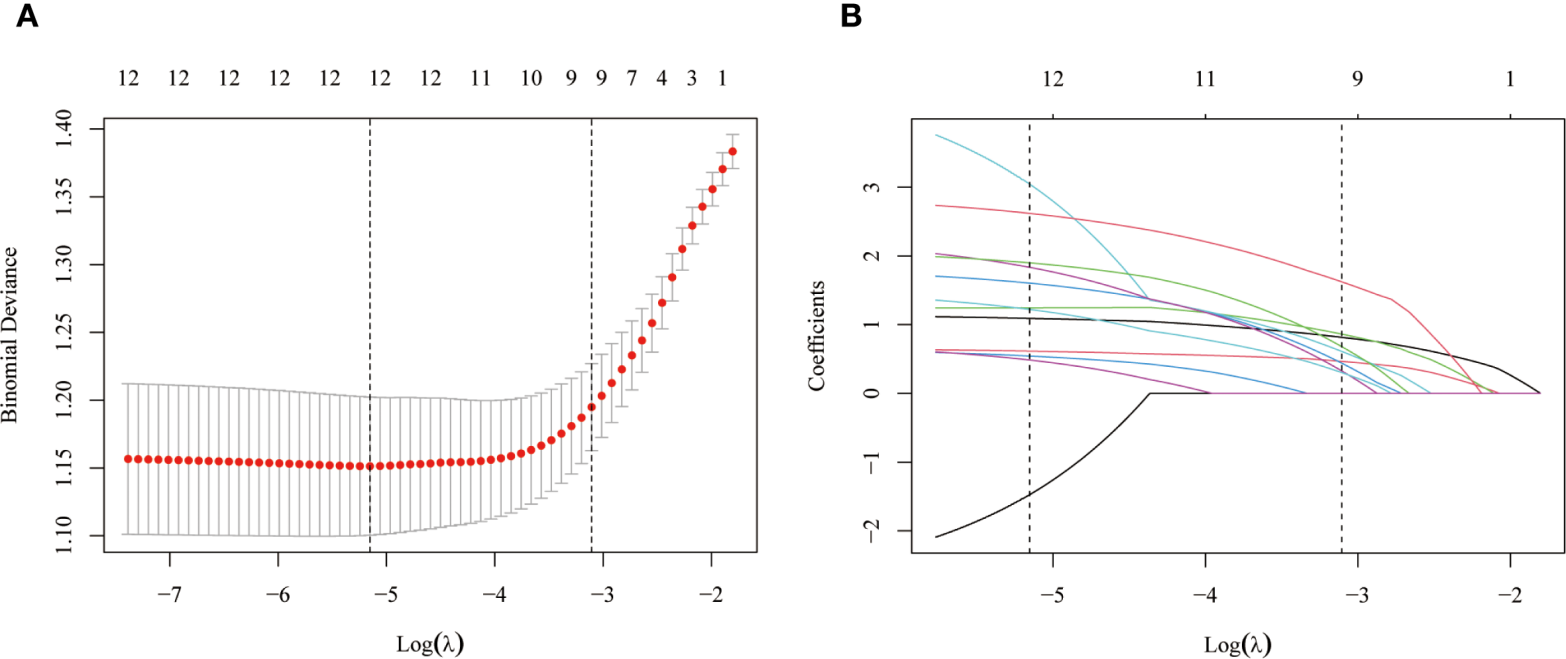
Feature selection using the Least Absolute Shrinkage and Selection Operator (LASSO) binary logistic regression model. **(A)** Selection of the optimal tuning parameter (λ) in the LASSO model using 10-fold cross-validation via minimum criteria. The dotted vertical lines represent the optimal values using the minimum criteria (left) and the 1-standard error criteria (right). **(B)** LASSO coefficient profiles of the 12 candidate clinical features. A coefficient profile plot was produced against the log(λ) sequence.

In the training set, variables demonstrating statistical significance (P < 0.05) in the univariate analysis (**Supplementary Table 3**) were initially subjected to LASSO regression to select features. This step was implemented to minimize multicollinearity and prevent model overfitting. Ten-fold cross-validation was used to determine the optimal regularization parameter (λ). Based on the 1-standard error criterion (λ_1se_), the optimal value was 0.0448. Consequently, nine potential predictors with nonzero coefficients were retained (**Figures 2A, B**): age, diabetes duration, history of DR, history of DKD, PP, HR, FPG, HbA1c, and the TyG index. Subsequently, these nine features were entered into a multivariate logistic regression analysis. As shown in **Table 2**, seven independent risk factors for DCAN were identified: age (OR = 1.033, P = 0.030), diabetes duration (OR = 1.048, P = 0.026), history of DR (OR = 3.314, P < 0.001), history of DKD (OR = 2.025, P = 0.034), HR (OR = 1.029, P = 0.014), FPG (OR = 1.174, P = 0.030), and HbA1c (OR = 1.143, P = 0.033). Accordingly, these seven variables were used to construct the final predictive model.

**Table 2:** Multivariate logistic regression analysis for identifying independent predictors of DCAN in the training set.

Variable	β (SE)	Wald Z		OR (95% CI)	P-value
Age	0.033 (0.015)	2.167	1.033	1.033 (1.003–1.065)	0.030
Diabetes duration	0.046 (0.021)	2.219	1.048	1.048 (1.005–1.091)	0.026
History of DR	1.198 (0.315)	3.801	3.314	3.314 (1.786–6.147)	<0.001
History of DKD	0.705 (0.333)	2.116	2.025	2.025 (1.053–3.891)	0.034
Heart rate	0.029 (0.012)	2.462	1.029	1.029 (1.006–1.053)	0.014
FPG	0.160 (0.074)	2.172	1.174	1.174 (1.016–1.357)	0.030
HbA1c	0.134 (0.063)	2.126	1.143	1.143 (1.010–1.293)	0.033

### 3.3 Construction and validation of prediction models

The seven identified independent predictors were integrated into four distinct machine-learning algorithms: LR, RF, XGBoost, and LightGBM. To ensure an optimal model configuration, hyperparameter optimization was conducted on the training set using a 5-fold cross-validation strategy.

**Figure 3:**
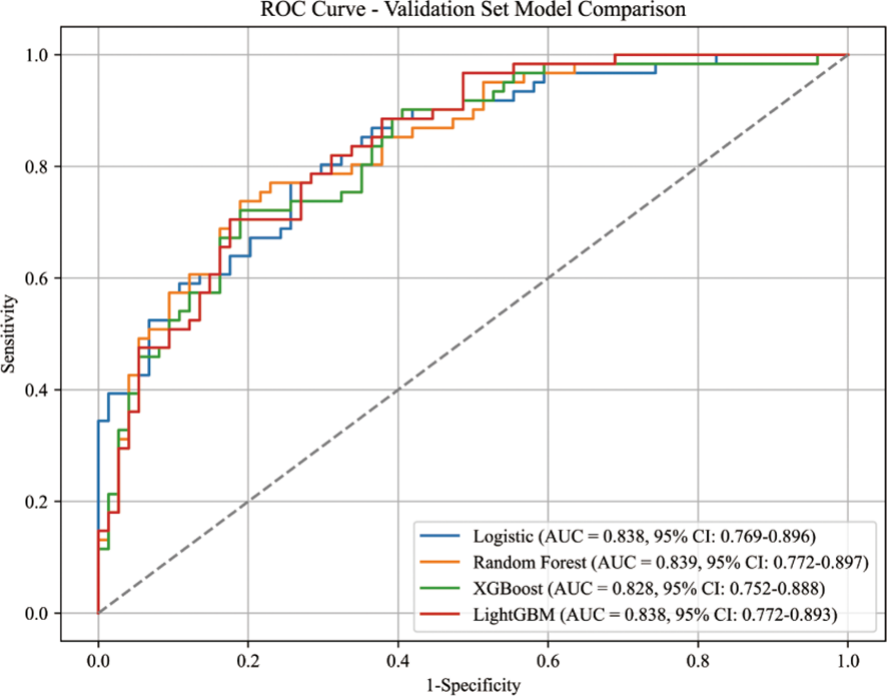
Comparison of receiver operating characteristic (ROC) curves among the four machine learning models in the validation set.

**Figure 4:**
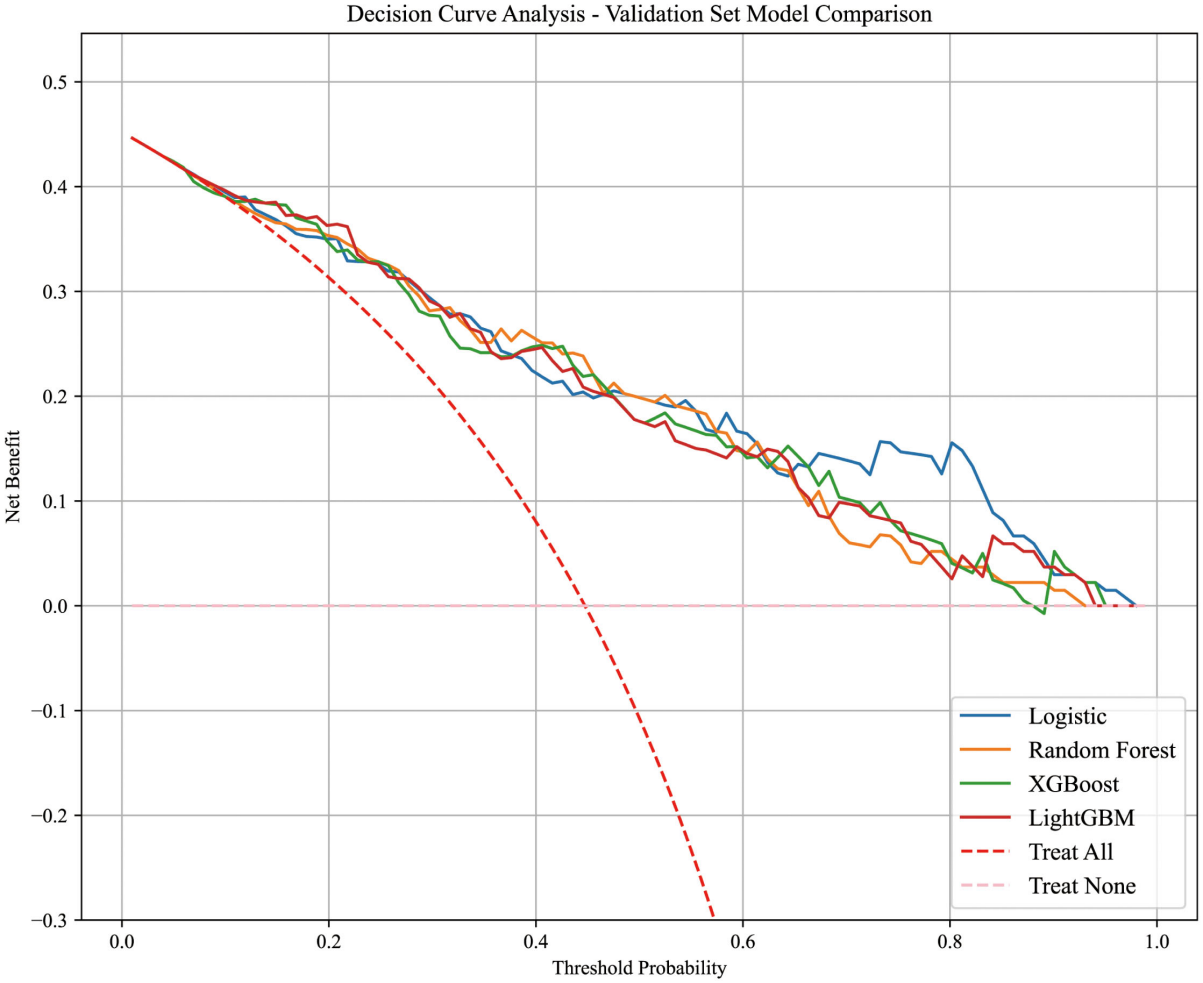
Decision curve analysis (DCA) evaluating the clinical net benefit of the four machine learning models in the validation set.

The comprehensive predictive performance of these models on the validation set is summarized in **Table 3**. As illustrated in **Figure 3**, all four methodologies demonstrated robust discriminative capabilities, with AUC values ranging from 0.828 to 0.839. Furthermore, DCA (**Figure 4**) indicated that all models provided substantial net clinical benefit relative to the “treat-all” or “treat-none” strategies.

**Table 3:** Predictive performance metrics of the four machine learning models in the validation set.

Model	AUC (95% CI)	Sensitivity	Specificity	PPV	NPV	Accuracy	F1 score
Logistic regression	0.838 (0.769–0.896)	0.770	0.743	0.712	0.797	0.756	0.740
Random forest	0.839 (0.772–0.897)	0.738	0.811	0.763	0.789	0.778	0.750
XGBoost	0.828 (0.752–0.888)	0.721	0.811	0.759	0.779	0.770	0.739
LightGBM	0.838 (0.772–0.893)	0.705	0.824	0.768	0.772	0.770	0.735

Although the AUC values were comparable across the algorithms, the LR model exhibited the most balanced performance profile and achieved the highest sensitivity (77.0%). Given the importance of sensitivity in screening and interpretability of linear models, the LR model was selected as the optimal candidate.

### 3.4 Interpretability and clinical application of the model

**Figure 5:**
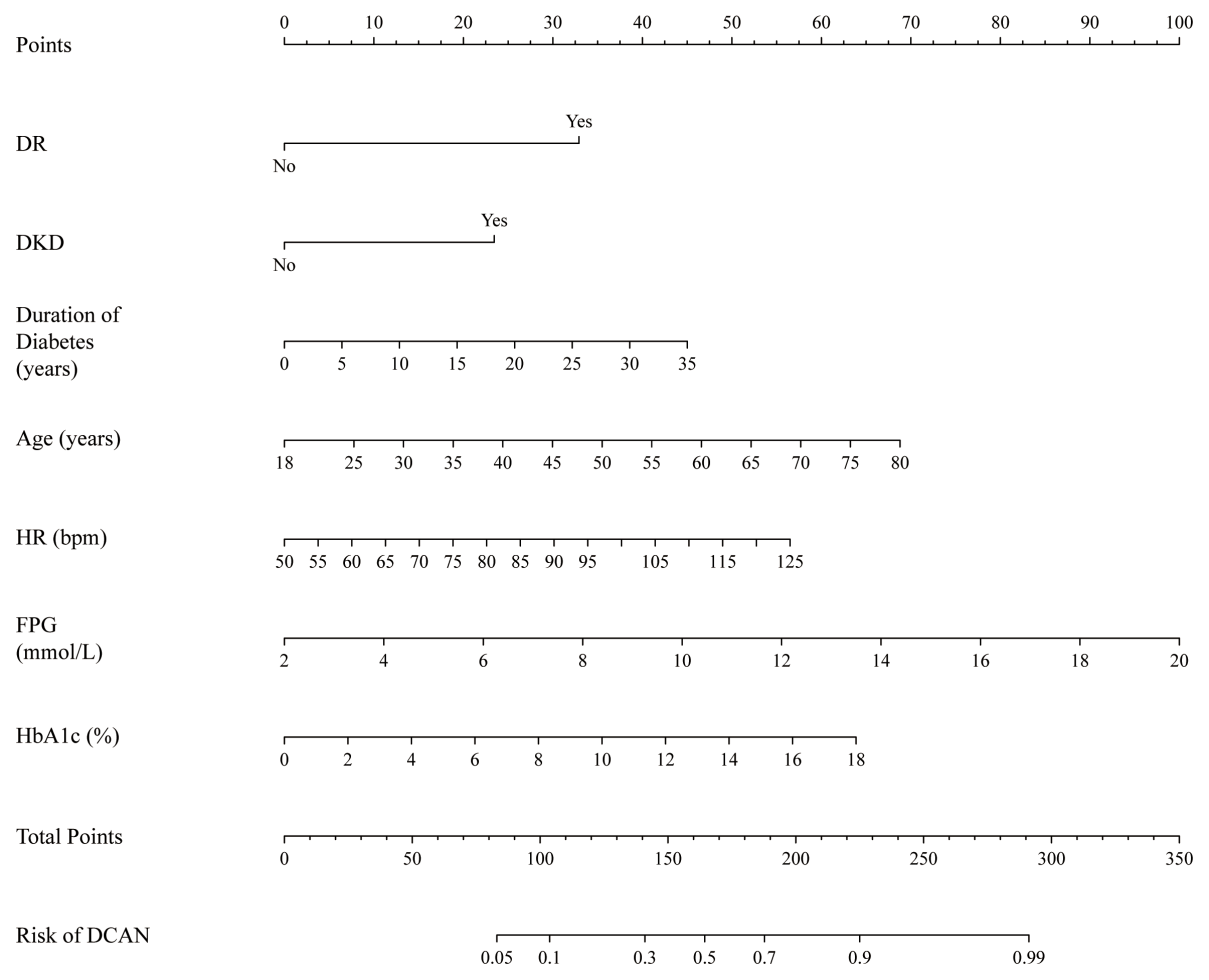
Nomogram for predicting the probability of DCAN based on history of DR, history of DKD, diabetes duration, age, heart rate (HR), fasting plasma glucose (FPG), and glycated hemoglobin (HbA1c).

**Figure 6:**
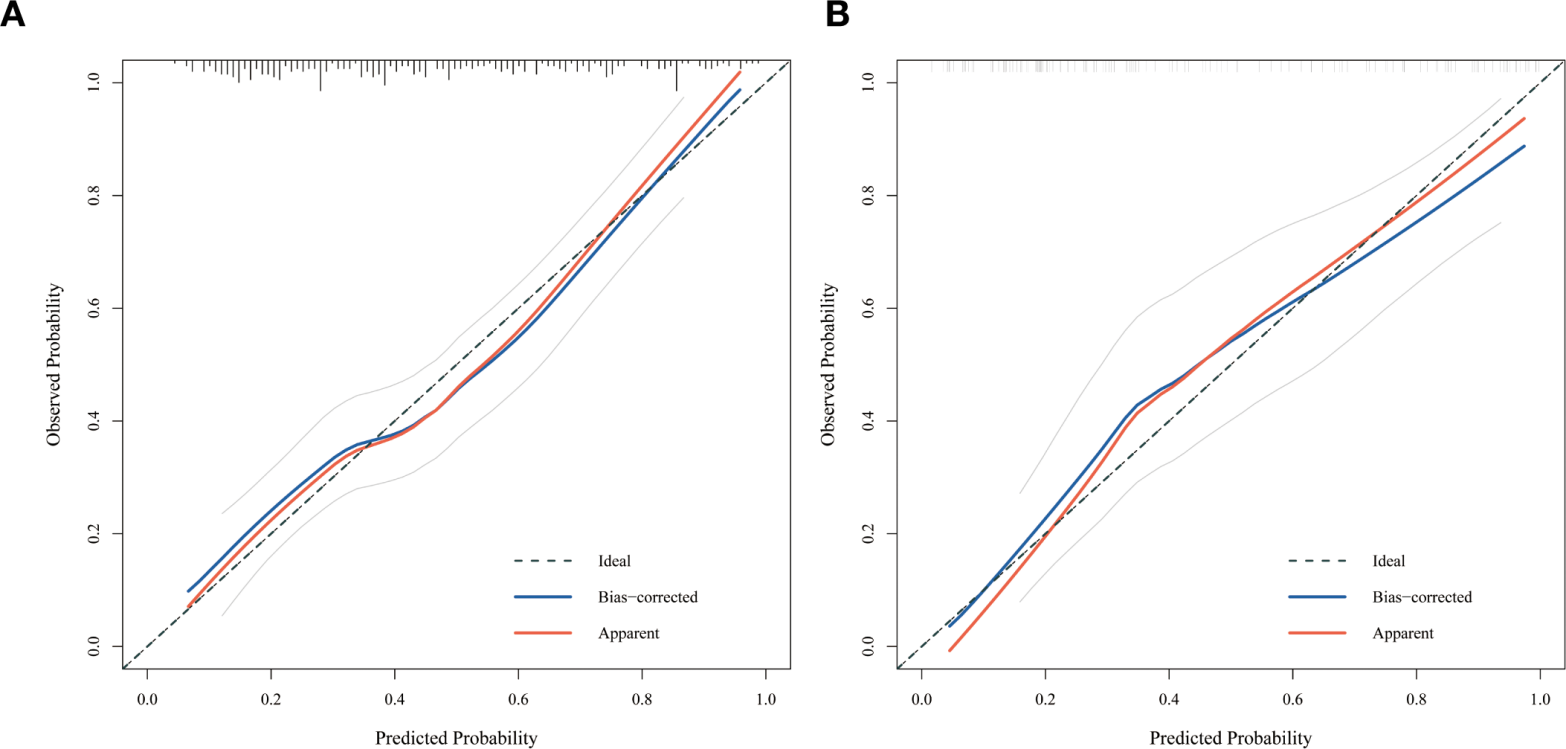
Calibration curves of the optimal Logistic Regression (LR) model. **(A)** Calibration in the training set. **(B)** Calibration in the validation set. The diagonal dotted line represents perfect prediction.

**Figure 7:**
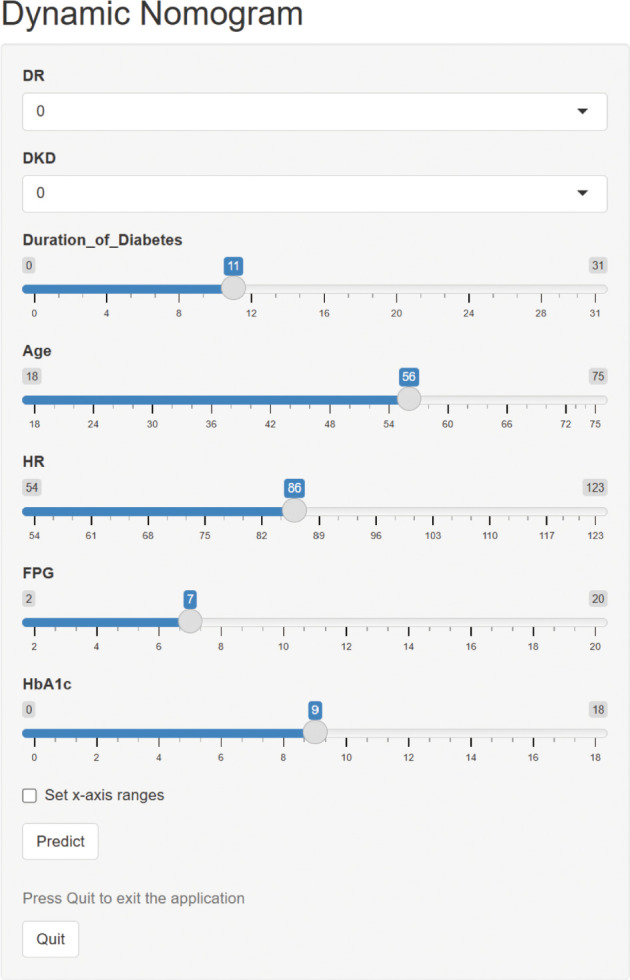
A web-based dynamic calculator for DCAN risk assessment. The calculator is accessible at: https://prediction-for-dcan.shinyapps.io/dynnomapp/.

A static nomogram was established based on the final logistic regression analysis to enhance the clinical utility and visualization of the predictive model (**Figure 5**). By summing the specific points assigned to each predictor, this tool enables an intuitive estimate of an individual’s probability of having DCAN. To validate the predictive accuracy of this nomogram, its calibration was assessed using the Hosmer–Lemeshow goodness-of-fit test and calibration curves. The results demonstrated non-significant P-values in both the training (χ^2^ = 12.364, df = 8, P = 0.136) and validation sets (χ^2^ = 5.775, df = 8, P = 0.672), indicating excellent agreement between the predicted probabilities and observed outcomes (**Figure 6**). To facilitate clinical deployment, we developed an interactive, web-based dynamic calculator using the Shiny framework (available at https://prediction-for-dcan.shinyapps.io/dynnomapp/) (**Figure 7**). This calculator enables clinicians to input specific parameters and obtain real-time risk predictions for DCAN, along with 95% CI.

## 4 Discussion

In this study, we developed and validated a prediction model for identifying the presence of DCAN in patients with diabetes using routinely available clinical variables. Through a combined feature selection strategy, seven predictors were retained: age, diabetes duration, history of DR, history of DKD, resting HR, FPG, and HbA1c. Among the four modeling approaches evaluated, the LR model provided the most balanced performance and interpretability, achieving an AUC of 0.838 and a sensitivity of 77.0% in the validation cohort. The final model was translated into a nomogram and web-based calculator to support individualized pre-test risk estimation.

Established autonomic assessment frameworks, including Ewing’s battery, Toronto consensus-based classification, and Bellavere’s score-based assessment, remain essential for confirmatory diagnosis and severity grading because they directly assess cardiovagal and sympathetic autonomic responses (17, 19, 21). However, their implementation requires standardized maneuvers, trained personnel, patient cooperation, dedicated testing conditions, and additional clinical time, making their widespread use as universal first-line screening tools challenging in routine diabetes care (22). Given that recent reviews have emphasized that CAN remains under-recognized despite its prognostic significance (23), a practical risk-stratification tool based on routine clinical variables may help prioritize patients for confirmatory autonomic testing. In this context, our proposed model is not intended to replace established diagnostic systems, but to improve pre-test risk enrichment and help prioritize patients for confirmatory CARTs. Patients with high predicted probabilities may be referred for formal autonomic testing, whereas those with lower predicted probabilities may continue routine follow-up and risk factor optimization. Nevertheless, this model cannot directly quantify autonomic reflex impairment or determine DCAN severity.

Compared with previous DCAN risk assessment studies, the present model emphasizes clinical accessibility and translation. Menduni et al. developed clinical risk scores based on routinely available variables; however, the study did not systematically compare multiple modeling algorithms or provide a nomogram/web-based calculator (13). Abdalrada et al. developed a machine-learning model to predict early CAN using Ewing’s test-related variables and clinical data; however, the model primarily relied on autonomic testing variables and was not translated into an individualized visual tool (14). Nedergaard et al. used supervised ensemble classification to characterize CAN severity and identify important features, but many influential features were beat-to-beat or autonomic reflex-derived measures (15). In contrast, our model was developed from routinely available clinical variables, compared via multiple modeling approaches within the same dataset, and translated into a nomogram and web-based calculator. These features may facilitate pre-test risk stratification and practical implementation in routine diabetes care.

The retained predictors collectively reflect three clinically interpretable dimensions: cumulative metabolic exposure, microvascular complication burden, and autonomic imbalance. Consistent with prior findings, both DR and DKD were identified as strong independent predictors of DCAN, supporting the “common soil” hypothesis in which microvascular complications share overlapping pathogenic pathways driven by chronic hyperglycemia, oxidative stress, and endothelial dysfunction (24). The observed association between DR and DCAN may reflect parallel neurodegenerative processes affecting both the neurovascular unit of the retina and cardiac autonomic fibers (25). The association between DKD and DCAN may also reflect shared neurovascular and hemodynamic pathways, including sympathetic activation, renin–angiotensin–aldosterone system involvement, renal microvascular injury, and cardiorenal-autonomic interactions. Recent cohort and review evidence further suggests that CAN is associated with DKD and accelerated kidney function decline, strengthening the rationale for including DKD as a clinically meaningful predictor in the present model (26–28).

Both HbA1c and FPG were retained in the final model, suggesting that both chronic glycemic exposure and fasting hyperglycemia contribute predictive information for DCAN. Specifically, HbA1c reflects chronic glycemic burden, whereas FPG captures basal hyperglycemia that may not be fully represented by average glycemic control (29–31). Previous studies have shown that glycemic instability and poor glycemic control are associated with CAN, and improvement in glycemic status may contribute to better neuropathy-related outcomes (32).

Diabetes duration and age further reflected the cumulative nature of DCAN risk (1, 23). Longer diabetes duration may indicate prolonged exposure to hyperglycemia-related oxidative stress and microvascular injury, both of which can contribute to progressive autonomic nerve damage (1, 11, 33). Aging may also affect autonomic regulation through physiological declines in autonomic modulation and cardiovascular reflex adaptability (34). Therefore, the association between age and DCAN should also be interpreted in the context of normal age-related variation in autonomic regulation. Recent normative data for CARTs and HRV measures further support the need for future studies to explore age-adjusted reference values or age-stratified screening frameworks for DCAN assessment (35).

Notably, resting HR was identified as a key physiological predictor in our model. From a physiological perspective, HR reflects the balance between sympathetic and parasympathetic modulation. In patients with diabetes, early parasympathetic dysfunction and relative sympathetic predominance may contribute to resting HR elevation and autonomic imbalance (7, 23). This autonomic imbalance may be linked to insulin resistance, oxidative stress, and microvascular dysfunction, thereby promoting the development and progression of DCAN (7, 23). In addition, elevated resting HR has been associated with an increased risk of cardiovascular events and all-cause mortality in patients with diabetes or high cardiovascular risk (36, 37).

Beyond resting HR, both CART- and HRV-derived parameters have been used as predictors or markers of cardiac autonomic dysfunction (14, 15, 38). CART-based indices, including deep-breathing HR response, the Valsalva ratio, and the 30:15 ratio, provide more direct physiological assessment of cardiovagal function, whereas HRV parameters such as SDNN and RMSSD may detect early autonomic modulation abnormalities and have shown diagnostic value for CAN in specific diabetic populations (38–40). In addition, HRV-defined CAN has been associated with subsequent cardiovascular disease in long-term follow-up, further highlighting the prognostic relevance of HRV-derived autonomic markers (41). However, these approaches require standardized recording, signal processing, and methodological consistency, whereas resting HR is routinely available during ordinary clinical assessment (35, 39). Thus, resting HR should not be viewed as a replacement for CARTs or HRV, but as a pragmatic surrogate marker that may help identify patients requiring further autonomic evaluation.

Potential confounding from comorbidities and cardiovascular medications was addressed at both the design and analysis levels. Patients with overt thyroid dysfunction or recent use of medications directly affecting HR, cardiac conduction, or autonomic reflexes were excluded to minimize CART-related measurement bias. In addition, hypertension and the use of ACEIs/ARBs and dihydropyridine calcium channel blockers were evaluated in the present cohort and were not significantly associated with DCAN; therefore, they were not retained in the final prediction model. However, residual confounding cannot be fully excluded because medication effects may vary according to subclass, dosage, treatment duration, adherence, and underlying cardiovascular indications.

This study also has several limitations. First, owing to its retrospective cross-sectional design and lack of longitudinal data, the temporal sequence of the identified associations could not be firmly established; therefore, the model should be interpreted as a prediction tool rather than evidence of causal relationships. Second, although CARTs were used as the diagnostic reference and standardized procedures were applied, residual variability related to patient cooperation, testing conditions, and retrospective data collection cannot be completely excluded. Third, the model was designed to identify the presence of DCAN rather than to predict DCAN severity; therefore, it should not replace CARTs-based diagnostic staging. Fourth, although hypertension and the use of ACEIs/ARBs and calcium channel blockers were systematically evaluated, the retrospective design precluded the collection of detailed treatment-level data, including specific drug subclasses, dosing regimens, treatment duration, and long-term adherence patterns. Future prospective studies with comprehensive medication reconciliation are needed to address this limitation. Fifth, the model currently lacks external validation; therefore, its generalizability to other geographic or ethnic cohorts remains to be confirmed. Finally, although the current model performs well, incorporating additional biomarkers in the future may help develop a more comprehensive screening tool for DCAN.

## 5 Conclusion

This study developed a DCAN risk-prediction model using routine, accessible clinical indicators. To facilitate personalized risk assessment and screening, the model was translated into an intuitive nomogram and interactive web-based tool. While the current model demonstrated strong performance, future multicenter prospective studies with external validation are needed to confirm its generalizability and clinical utility across diverse populations.

## Data Availability

The raw data supporting the conclusions of this article will be made available by the authors, without undue reservation.
