# Supplementary material for: Development and validation of a nomogram model for predicting cardiac autonomic neuropathy in patients with diabetes
Source: Front Endocrinol (Lausanne). 2026 Jun 5;17:1831010. doi: 10.3389/fendo.2026.1831010 (PMC13278940; doi:10.3389/fendo.2026.1831010)
Supplement: Supplementary file 1 [file Table1.docx]

Supplementary Material

# Supplementary Table S1

Scoring criteria for cardiovascular autonomic reflex tests (CARTs).

| Test | Normal  (0 points) | Borderline  (1 point) | Abnormal  (2 points) |
| --- | --- | --- | --- |
|  | | | |
| Heart rate response to deep breathing, beats/min | ≥15 | 11–14 | ≤10 |
| Valsalva ratio | ≥1.21 | 1.11–1.20 | ≤1.10 |
| 30:15 test | ≥1.04 | 1.01–1.03 | ≤1.00 |
|  | | | |
| Orthostatic systolic blood pressure change, mmHg | ≤10 | 11–29 | ≥30 |

# Supplementary Table S2.

Final selected tuned hyperparameters for RF, XGBoost, and LightGBM. Only hyperparameters tuned in the grid search are shown; other parameters were kept at their default settings.

| Model | Final hyperparameters |
| --- | --- |
| Random Forest | n_estimators = 240; max_features = sqrt |
| XGBoost | learning_rate = 0.1; max_depth = 3; n_estimators = 50; subsample = 1.0 |
| LightGBM | colsample_bytree = 0.8; learning_rate = 0.05; max_depth = 3; min_child_samples = 20; n_estimators = 80; num_leaves = 15; reg_alpha = 0.1; reg_lambda = 0.5; subsample = 0.7 |

# Supplementary Table S3.

# Univariate analysis of potential risk factors for DCAN in the training set. Only variables with P < 0.05 are shown.

| Variables | Non-DCAN (n=175) | DCAN (n=143) | P-value |
| --- | --- | --- | --- |
| Age, years | 55.00 [48.00, 60.50] | 60.00 [53.00, 65.00] | <0.001 |
| Diabetes duration, years | 8.00 [3.00, 14.00] | 11.00 [6.00, 20.00] | <0.001 |
| History of DR | 25 (14.29) | 63 (44.06) | <0.001 |
| History of DKD | 22 (12.57) | 51 (35.66) | <0.001 |
| SBP, mmHg | 126.00 [116.00, 135.00] | 130.00 [115.00, 143.00] | 0.028 |
| Pulse pressure, mmHg | 43.00 [35.00, 52.00] | 50.00 [39.50, 59.50] | <0.001 |
| Heart rate, bpm | 84.85 (11.82) | 88.10 (12.43) | 0.017 |
| FPG, mmol/L | 6.31 [5.07, 7.70] | 7.36 [5.94, 9.43] | <0.001 |
| HbA1c, % | 8.10 [6.90, 9.75] | 9.10 [7.60, 10.80] | 0.001 |
| UACR, mg/g | 5.96 [3.09, 14.53] | 16.35 [6.73, 69.88] | <0.001 |
| TyG index | 8.88 [8.36, 9.38] | 9.14 [8.70, 9.61] | 0.001 |
| SIRI | 0.80 [0.54, 1.14] | 0.90 [0.66, 1.32] | 0.022 |

Note: Data are presented as n (%), mean ± SD, or median [IQR], as appropriate. P-values were calculated using Student’s t-test or the Mann–Whitney U test for continuous variables and the chi-square test or Fisher’s exact test for categorical variables, as appropriate. Only variables with P < 0.05 in the univariate analysis of the training set are shown. DCAN, diabetic cardiac autonomic neuropathy; DR, diabetic retinopathy; DKD, diabetic kidney disease; FPG, fasting plasma glucose; HbA1c, glycated hemoglobin; SBP, systolic blood pressure; UACR, urinary albumin-to-creatinine ratio; TyG, triglyceride-glucose index; SIRI, systemic inflammation response index.
